# Faith, Meaning, and Life After Kidney Transplant Failure in Türkiye: A Qualitative Study of Spiritual Coping and Adjustment to Dialysis

**DOI:** 10.1007/s10943-026-02707-1

**Published:** 2026-06-24

**Authors:** Deniz Tasdemir, Hatice Ceylan

**Affiliations:** Department of Nursing, Bucak School of Health, Burdur Mehmet Akif Ersoy University, 15300 Campus, Burdur, Turkey

**Keywords:** Spirituality, Religious coping, Quality of life, Kidney transplant failure, Dialysis, Meaning-making, Chronic illness, Qualitative research

## Abstract

Kidney transplant failure and the subsequent return to dialysis constitute a profound biographical disruption that extends beyond physical decline to encompass existential and spiritual challenges. While the impact of graft failure on quality of life has been documented, less is known about how individuals draw on spiritual and religious resources to make sense of this transition. This qualitative study explores how patients reconstruct quality of life following kidney transplant failure, with particular attention to spiritual coping and meaning-making processes. Using a descriptive qualitative design, semi-structured interviews were conducted with 21 adults receiving hemodialysis or peritoneal dialysis after transplant failure in Türkiye. Data were analyzed using reflexive thematic analysis following Braun and Clarke’s approach. Analysis revealed four interrelated themes. Participants described intense emotional distress, uncertainty, and loss. Spirituality functioned both as a source of comfort and as a means of reconstructing meaning, enabling participants to reframe suffering, sustain hope, and renegotiate their sense of self and future. Social and familial support further reinforced these processes. The findings suggest that quality of life after transplant failure is shaped not only by physical and psychosocial factors but also by spiritual beliefs and practices. Integrating spiritual care into clinical settings may enhance holistic, multidisciplinary support for patients undergoing this challenging transition.

**Supplementary Information:**

The online version contains supplementary material available at https://doi.org/10.1007/s10943-026-02707-1.

## Introduction

Chronic kidney disease (CKD) represents a substantial and growing global public health challenge, currently affecting approximately 10% of the adult population globally (Kalantar-Zadeh et al., 2021). For individuals whose condition progresses to end-stage renal disease, kidney transplantation is considered the gold standard of treatment because it increases life expectancy and improves quality of life by eliminating the stringent restrictions of dialysis (Güzel et al., 2024). However, this “new lease on life” is often precarious, as approximately 1 in 5 recipients experience graft loss within 5 years of the procedure, and over half will fail within 10 years (Slominska et al., 2024). Graft failure constitutes a profound “biographical disruption,” plunging patients back into an existential crisis marked by a sudden loss of control, hope, and medical certainty (Elias et al., 2025; Slominska et al., 2024).

Despite the catastrophic nature of this disruption, current clinical approaches often prioritize biomedical metrics over the patient’s subjective “lived experience” (Slominska et al., 2024). This transition is frequently characterized as a “dark postoperative recovery stage” where recipients face significant physiological, psychological, and social difficulties upon returning home (Yang et al., 2020). Spirituality and religion have emerged as vital but underexplored resources that patients utilize to navigate these traumatic health transitions and maintain psychological equilibrium (Bravin et al., 2019; Güzel et al., 2024). Given Türkiye’s unique cultural and religious landscape, understanding these dimensions is essential for providing holistic care. This study aims to qualitatively explore the spiritual coping mechanisms and meaning-making processes of patients in Türkiye adjusting to maintenance dialysis following kidney transplant failure. Accordingly, the guiding research question was: “How do patients in Türkiye experience and utilize spiritual and religious resources to reconstruct meaning and quality of life after kidney transplant failure?”.

## Literature Review

Spirituality and religious attitudes play a central role in how individuals interpret and respond to the suffering imposed by chronic illness (Güzel et al., 2024). For many patients undergoing renal replacement therapy, spiritual beliefs serve as a “lifeline,” providing hope and facilitating adjustment to life-sustaining technology (Elias et al., 2025). Seminal research has long established that religious involvement is positively related to mental health indicators and can provide essential protection against the psychological distress associated with long-term illness (Koenig et al., 2001). Furthermore, a positive religious attitude can transform stressful clinical events into opportunities for personal growth and internal peace (Güzel et al., 2024).

The experience of transplant failure is particularly traumatic, often described by recipients as the loss of a previous life and an imagined future (Slominska et al., 2024). Research indicates that patients who have experienced the freedom of a functioning transplant view the return to dialysis as more debilitating than those who have never had a transplant (Nahamin et al., 2020). This transition involves a complex range of tangible and experiential losses, including the loss of the transplant team as a primary support system and a diminished sense of identity (Slominska et al., 2024). Patients must navigate a “trajectory of uncertainty” where the constant threat of death and the rigors of dialysis treatment significantly impair their quality of life (Elias et al., 2025).

Two primary theoretical frameworks are instrumental in understanding this adjustment process: Pargament’s theory of religious coping and Park’s meaning-making model. Pargament et al., (1998) distinguish between positive religious coping, which involves seeking spiritual support and finding benevolent meaning in illness, and negative religious coping, which may manifest as feelings of abandonment by the divine. Although negative religious coping was not a prominent theme in the present study, it remains an important dimension, as it has been associated with poorer psychological adjustment in chronic illness contexts. Complementing this, Park’s, (2010) meaning-making model posits that chronic illness disrupts an individual’s “global meaning,” necessitating a process of “situational meaning-making” to reconcile the trauma of illness with one’s core beliefs. In religious contexts, patients often utilize multilateral spiritual approaches, such as viewing illness as a “God’s examination” or a predetermined destiny, to retrieve lost peace (Nahamin et al., 2020).

Despite these theoretical insights, a clear research gap persists in the current literature. Most existing studies are quantitative and cross-sectional, often failing to capture the depth and complexity of the post-failure experience (Güzel et al., 2024). Furthermore, there is a notable lack of qualitative research focusing specifically on transplant failure as a distinct phenomenon from native kidney failure (Slominska et al., 2024). Finally, while Türkiye possesses a unique cultural and religious environment, there is a dearth of culturally contextualized qualitative data exploring how spiritual meaning-making facilitates adjustment to dialysis after the devastating loss of an allograft. Addressing these gaps is critical for developing supportive techniques that enhance recovery in kidney transplant recipients (Nahamin et al., 2020). This study specifically addresses this gap by providing an in-depth qualitative exploration of spiritual coping and meaning-making processes among patients in Türkiye adjusting to dialysis following kidney transplant failure.

## Methods

### Study Design

This study employed a descriptive qualitative design to explore the psychosocial adjustment of patients returning to dialysis following kidney transplant failure. A qualitative approach was selected to gain in-depth insight into participants’ experiences, meanings, and adaptive processes during this transition. In this sense, this study focuses on how individuals interpret and give meaning to life events, rather than imposing pre-established theoretical frameworks (Taşdemir, 2022). This design was particularly well-suited given the sensitive nature of the topic and the need to capture the nuanced impact of transplant rejection and resuming dialysis on patients’ lives. This study was designed and reported in accordance with the Consolidated Criteria for Reporting Qualitative Research (COREQ).

### Setting

The study was conducted in Antalya, a province in Türkiye that actively provides kidney transplantation and dialysis services. This setting was selected because it includes tertiary hospitals and specialized nephrology centers, thereby providing access to patients who had undergone kidney transplantation and later returned to dialysis. The inclusion criteria for participation were being 18 years or older, having experienced kidney transplant rejection, currently undergoing dialysis treatment, being able to communicate effectively in Turkish, and being willing to share personal experiences. Exclusion criteria included severe cognitive or psychiatric disorders that could hinder participation in an in-depth interview.

The recruitment process yielded 21 participants, including both men and women across a diverse age range. Data collection continued until saturation was achieved, meaning that no new codes or themes emerged despite the inclusion of new participants. Data saturation was reached after the 21st interview, confirming that the identified themes were sufficient to represent the phenomenon under investigation (Taşdemir & Taşdemir, 2026; Yang et al., 2022).

### Sampling

A snowball sampling method was employed for participant recruitment. This approach is frequently utilized in qualitative research when working with populations that are difficult to access, particularly in studies involving sensitive health-related issues such as organ transplantation and rejection. Initial participants were identified through nephrology units and dialysis centers, and subsequent participants were referred by individuals who had already taken part in the study. Recruitment continued in this manner until data saturation was reached. Snowball sampling facilitated the identification of participants who shared similar experiences but who might not otherwise have been easily reachable through conventional recruitment strategies (Leighton et al., 2021). However, it is acknowledged that this sampling approach may introduce selection bias, as participants are more likely to refer individuals with similar characteristics or perspectives.

### Data collection

Data were collected between December 2024 and May 2025 through semi-structured interviews conducted via a secure online platform (Zoom), ensuring privacy and confidentiality throughout the process. All interviews followed the same interview guide, regardless of the delivery mode. Semi-structured interviewing was chosen because it ensures key topics are covered while allowing participants to elaborate on their personal experiences (Kallio et al., 2016). The interview guide (Online Appendix 1: Interview guide) was developed based on an extensive literature review and refined through consultation with qualitative research experts and a nephrology specialist (Kułakowska et al., 2023; Slominska et al., 2024).

A brief rapport-building session was held with each participant before the interview to foster trust and openness. Participants were informed that the interviewer was a nurse researcher with professional experience in nephrology care and that the purpose of the interview was to understand their lived experiences rather than evaluate their clinical condition. Questions encouraged participants to describe their emotions following rejection, their adaptation to dialysis, changes in daily life, social and family roles, coping mechanisms, and their outlook toward the future. Each participant took part in a single interview; no repeat interviews were conducted.

Individual interviews were conducted via an online platform with participants who had provided informed consent to participate in the study. Measures were implemented to ensure the privacy and confidentiality of all participants. Interviews lasted 45–75 min and were conducted in Turkish by the first author, who had prior training and experience in qualitative interviewing. All interviews were audio-recorded with participants’ consent, and detailed field notes were taken to capture contextual details and non-verbal expressions. Transcripts were produced verbatim, and all identifying information was removed during the transcription process to ensure anonymity.

### Data analysis

Data were analyzed using the reflexive thematic analysis framework described by Braun and Clarke, (2006), which provides a structured yet flexible approach for identifying and interpreting patterns in qualitative data (Braun & Clarke, 2006). The process began with repeated readings of the transcripts to ensure familiarity with the data. Initial codes were generated line by line and collated systematically. Coding was primarily inductive, enabling themes to be grounded in participants’ narratives, while remaining sensitized to relevant theoretical frameworks. These codes were then organized into potential themes, which were reviewed and refined to ensure coherence and distinctiveness. Themes were clearly defined and named, reflecting participants’ shared meanings and unique experiences. The analysis was supported by the use of NVivo 12 software (QSR International), which allowed systematic organization and retrieval of data. Coding was undertaken by two researchers to facilitate deeper engagement with the data. In line with the principles of reflexive thematic analysis, this process was not aimed at achieving inter-coder reliability; rather, it supported reflexive dialogue and critical interpretation. Themes were developed through iterative discussions and collaborative reflection within the research team. Direct quotations from participants were selected to illustrate each theme, thereby grounding interpretations in the original data and preserving the authenticity of participants’ voices.

## Researcher’s Role and Reflexivity

The first author, who conducted all interviews, had prior professional experience in nephrology nursing. This background provided valuable contextual understanding but also posed potential bias. To minimize this influence, reflexivity was maintained throughout the study. The researcher kept a reflective journal to document personal assumptions, emotional reactions, and decisions made during data collection and analysis. Reflexive notes were reviewed alongside peer debriefing sessions to ensure that interpretations remained grounded in participants’ narratives rather than the researcher’s preconceptions (Olmos-Vega et al., 2023).

### Rigor

The rigor of the study was ensured by applying the criteria proposed by Lincoln and Guba, (1985), which continue to be widely used for evaluating trustworthiness in qualitative research (Enworo, 2023). Credibility was enhanced through prolonged engagement with participants, triangulation of data sources (interview transcripts and field notes), and peer debriefing within the research team. To further strengthen credibility, participants were invited to review and provide feedback on key findings and thematic summaries via online communication, ensuring that interpretations accurately reflected their experiences. Dependability was supported by maintaining a detailed audit trail documenting all methodological and analytical decisions. Confirmability was established through reflexive journaling and independent coding by two researchers using NVivo 12. Transferability was achieved by providing thick descriptions of the research context, participants, and settings, allowing readers to assess the relevance of the findings to other contexts.

### Ethical Considerations

Ethical approval was obtained from the Ethics Committee of Local University (Decision No: GO 2024/648, Date: 05.11.2024). All participants were informed about the purpose of the research, the voluntary nature of their participation, and their right to withdraw at any time without any negative consequences for their medical care. Informed consent was obtained from each participant prior to the interviews. Confidentiality was maintained by anonymizing transcripts and using pseudonyms. Audio files and transcripts were stored on password-protected devices accessible only to the research team. The study was conducted in line with the ethical principles outlined in the Declaration of Helsinki.

## Results

A total of 21 individuals participated in the study, including 12 men and 9 women, aged 35–67 years. Most participants were married and receiving hemodialysis, while a smaller number were undergoing peritoneal dialysis. The duration of dialysis following graft failure ranged from 6 months to 12 years. Detailed demographic and clinical characteristics are presented in Table 1.

**Table 1:** Demographic and clinical characteristics of participants (*N* = 21)

Variable	n	%
*Gender*		
Male	12	57.1
Female	9	42.9
*Age (years)*		
20–39	7	33.3
40–59	10	47.6
≥ 60	4	19.1
*Marital status*		
Married	15	71.4
Single/Divorced/Widowed	6	28.6
*Type of dialysis*		
Hemodialysis	16	76.2
Peritoneal dialysis	5	23.8
*Time on dialysis (after rejection)*		
≤ 2 years	8	38.1
3–5 years	7	33.3
≥ 6 years	6	28.6

Analysis of the data revealed four interrelated themes that describe the multidimensional process through which participants experienced and reconstructed quality of life after kidney transplant failure: (1) Experiencing Disruption: Emotional and Existential Distress, (2) Embodied Burden and Social Dislocation, (3) Spiritual Coping and Meaning-Making as Pathways to Endurance, and (4) Reconstructing Hope, Identity, and Future Through Faith and Adaptation. These themes, along with their categories and subthemes, are summarized in Table 2.

**Table 2:** Themes, categories, and subthemes

Themes	Category	Subthemes
Experiencing disruption	Emotional shock	Denial, fear, grief, loss of control
	Psychological distress	
Embodied burden and social dislocation	Physical strain	Fatigue, dependency, role loss, financial strain
	Social life disruption	
Spiritual coping and meaning-making	Faith-based coping	Trust in God, prayer, acceptance, illness as test/destiny
	Meaning reconstruction	
Reconstructing hope and identity	Identity transformation	New normal, hope for transplant, uncertainty
	Future orientation	

### Theme 1: Experiencing Disruption: Emotional and Existential Distress After Graft Failure

Participants described transplant failure as a profound disruption that extended beyond physical decline to deeply affect their emotional and existential worlds. The initial response was often characterized by shock and disbelief, reflecting the collapse of expectations associated with transplantation. One participant explained, “*When they told me my kidney had failed, I couldn’t believe it. I thought there must be a mistake. After all I had gone through, it felt like my world collapsed*” (Participant 6).

Beyond the initial shock, participants expressed intense emotional distress, including fear, sadness, and hopelessness. These reactions were frequently intertwined with existential concerns about dependency, loss of autonomy, and an uncertain future. As one participant noted, “*I am back to the machine again. I worry constantly about depending on my children. I feel like I am taking away their freedom*” (Participant 11).

For some, transplant failure represented not only a medical setback but a disruption of life meaning and anticipated life trajectories. A younger participant reflected, “*I was only 30 when my transplant failed. I thought my life was over. Sometimes I just wanted to stay in bed and not face the day*” (Participant 3). These narratives highlight how graft failure constitutes a “secondary life disruption,” marked by both psychological distress and existential uncertainty.

### Theme 2: Embodied Burden and Social Dislocation in Returning to Dialysis

Participants described returning to dialysis as an embodied experience characterized by physical exhaustion and ongoing treatment demands. Fatigue was one of the most frequently reported challenges, often limiting daily functioning and reducing overall quality of life. One participant stated, “*Dialysis drains me completely. After every session, I feel like I don’t have any energy left*” (Participant 9).

The physical burden of dialysis was closely linked to disruptions in social roles and relationships. Many participants reported difficulties in maintaining family responsibilities and social engagement. A participant shared, “*I used to take care of my grandchildren. Now I can’t even walk them to the park because I’m always tired or at the hospital*” (Participant 13).

In addition, financial strain emerged as a significant concern, often due to reduced work capacity or job loss following graft failure. One participant explained, “*Before rejection, I had returned to work. Now I had to quit again, and financially, it’s very hard on us*” (Participant 7). These findings illustrate how the return to dialysis disrupts not only the body but also social identity and economic stability.

### Theme 3: Spiritual Coping and Meaning-Making as Pathways to Endurance

Spirituality emerged as a central dimension through which participants coped with and interpreted their experiences of transplant failure and dialysis. Rather than functioning solely as a supportive resource, spiritual beliefs provided a broader framework for understanding suffering, uncertainty, and life transitions.

Many participants described entrusting their situation to God as a way to manage emotional distress and regain a sense of stability. This reliance on faith often involved surrender, acceptance, and trust in a higher power. As one participant expressed, “*I leave everything to God. That is what keeps me going. Without faith, I would have given up a long time ago*” (Participant 15).

Meaning-making processes were evident in participants’ reinterpretations of transplant failure in spiritual or existential terms. Some framed their illness as a test, destiny, or part of a divine plan, which helped them integrate the experience into a meaningful narrative. For example, one participant stated, “*Maybe this is a test for me. I try to be patient and accept what God has given*” (Participant 10).

Spiritual coping was further supported by relational contexts, including family encouragement and shared religious practices. Participants emphasized that these connections reinforced their faith and reduced feelings of isolation. As one individual noted, “*When I pray and talk with my family, I feel stronger. It reminds me that I am not alone in this*” (Participant 12).

Overall, spirituality functioned as both an emotional anchor and a meaning-making system, enabling participants to endure ongoing challenges and sustain a sense of coherence in their lives.

### Theme 4: Reconstructing Hope, Identity, and Future Through Faith and Adaptation

Despite experiencing significant distress, participants described ongoing efforts to reconstruct their sense of identity and redefine what constituted a meaningful life. This process often involved integrating dialysis into a “new normal” and adjusting expectations accordingly. One participant reflected, “*I had to accept that my life is different now. Dialysis is not the end, it’s just another way of living*” (Participant 8).

Hope emerged as a dynamic and evolving construct. Some participants maintained hope for retransplantation, while others focused on maintaining daily functioning and emotional stability. However, hope was often accompanied by uncertainty and ambivalence. As one participant explained, “*I still dream of another transplant, but I am also afraid of going through the same rejection again*” (Participant 20).

For others, the future remained uncertain and difficult to envision. One participant stated, “*The future is a big question mark for me. I don’t know how long I can carry on like this*” (Participant 5). However, some participants navigated this uncertainty through faith-based meaning-making. As one individual expressed, “*I don’t know what the future holds, but I believe there is a reason for everything. That belief gives me strength to continue*” (Participant 4).

## Discussion

This study contributes to the literature by providing culturally contextualized qualitative insights into the role of spirituality in adjusting to dialysis after graft failure. The findings of this study underscore that kidney transplant failure is not merely a clinical regression but a profound “biographical disruption” that necessitates a comprehensive reconstruction of the self (Elias et al., 2025; Slominska et al., 2024). Participants described the transition from a “medical marvel” post-transplant to an “unbelievable trauma” following graft loss, illustrating an existential crisis where spirituality emerges as the primary tool for endurance (McKeaveney et al., 2022; Slominska et al., 2024). The pervasive themes of shock, dependency, and existential distress reveal that for these patients, the return to dialysis is often perceived as more debilitating than the initial diagnosis, echoing the “living numbness” reported in other post-failure populations (McKeaveney et al., 2022; Nahamin et al., 2020).

Interpreting these results through Park’s, (2010) meaning-making model, it is evident that the catastrophic loss of an allograft severely disrupts a patient’s “global meaning” their core beliefs about health, predictability, and the future. To reconcile this trauma, participants engaged in “situational meaning-making,” utilizing spiritual frameworks to bridge the gap between their prior expectations and their current reality (Park, 2010; Slominska et al., 2024). This process is further explained by Pargament’s theory of religious coping, in which reliance on faith serves as a “lifeline” that transforms stressful events into opportunities for spiritual growth and internal peace (Güzel et al., 2024; Pargament et al., 1998). Participants predominantly utilized positive religious coping, which has been associated with higher levels of hope and better renal outcomes in chronic illness settings (Bravin et al., 2019; Pargament et al., 1998). Although positive religious coping was predominant in the present study, the theoretical possibility of negative religious coping, such as feelings of spiritual discontent or perceived abandonment by God, should also be acknowledged, as highlighted in Pargament’s framework, even though it was not a prominent feature of participants’ narratives.

The manifold losses reported in this study, including loss of control, certainty, and quality of life, align with recent qualitative evidence suggesting that patients lose “much more than a transplant” (Slominska et al., 2024). Similar to recipients in other contexts, our participants ruminate on death and experience a sudden “social dislocation” upon losing the specialized support of the transplant team (McKeaveney et al., 2022; Slominska et al., 2024). However, a unique finding in this study is the high degree of spiritual “re-correcting” where participants actively reshaped their identity by viewing dialysis not as a failure, but as a divine “medical marvel” or a second opportunity provided by God (McKeaveney et al., 2022; Yang et al., 2020).

The interpretation of illness as a “God’s examination” or predetermined destiny was a pivotal finding, providing participants with a powerful refuge to seek peace (Nahamin et al., 2020). This spiritual approach, rooted in the Islamic concepts of *kismet* (fate) and *sabr* (patience), allows individuals to surrender to a higher power, thereby alleviating the psychological burden of self-blame often associated with graft failure (Güzel et al., 2024; Nahamin et al., 2020). Seminal research supports that such religious involvement acts as a vital protective factor against the anxiety and depression that typically permeate the first few months of renal replacement therapy (Koenig et al., 2001; Nahamin et al., 2020).

Clinical implications of this study suggest that nephrology practice must move beyond purely biomedical models to incorporate “in-house” psychosocial and spiritual care (McKeaveney et al., 2022; Slominska et al., 2024). Healthcare professionals should be aware that spiritual needs are an integral part of the “lived experience” of transplant failure and that addressing these needs can foster healthy adjustment and personal resilience (Bravin et al., 2019; Güzel et al., 2024). In addition, interventions that strengthen family and social support networks may further enhance resilience and psychosocial adaptation among dialysis patients (Karami et al., 2025). The role of the renal social worker and multidisciplinary team is critical in facilitating these “difficult conversations” and supporting patients as they navigate the dark postoperative recovery stage (Hansen et al., 2021; McKeaveney et al., 2022; Yang et al., 2020).

In the cultural context of Türkiye, deep devotion to religious beliefs significantly enhances the quality of life and adherence to treatment regimens (Güzel et al., 2024). Faith does not merely function as a coping mechanism but as a comprehensive meaning system that helps patients interpret suffering as part of a divine plan (Güzel et al., 2024; Nahamin et al., 2020). Understanding this culturally contextualized meaning-making is essential for providing patient-centered care that respects the individual’s worldview and promotes psychological well-being (Güzel et al., 2024; Park, 2010).

## Limitations

This study has several limitations that should be considered when interpreting the findings. Although data saturation was achieved with 21 participants, the sample size may not capture the full range of experiences of patients returning to dialysis after kidney transplant rejection. Additionally, participants who were willing to share their experiences may represent individuals with higher emotional awareness or coping capacity, potentially introducing self-selection bias. As the study was conducted in a single geographic setting, the transferability of the findings to other cultural or regional contexts may be limited. Finally, the findings are based on subjective self-reports and may not fully reflect the perspectives of healthcare professionals or family members involved in patients’ care. Future studies using longitudinal or multi-perspective designs could provide a more comprehensive understanding of the long-term psychosocial adaptation process following graft failure.

## Conclusion

In conclusion, kidney transplant failure represents a profound biographical and existential disruption that extends beyond physical health to affect patients’ sense of identity, meaning, and future orientation. The findings of this study highlight spirituality as a central resource through which individuals interpret suffering, reconstruct meaning, and sustain hope as they adjust to life on dialysis. Through faith-based coping and meaning-making, participants navigated uncertainty, redefined their sense of self, and established a “new normal.” These insights underscore the importance of integrating culturally sensitive, multidisciplinary approaches that include spiritual care into nephrology practice to better support patients during this challenging transition. The findings of this study identify spiritual coping and meaning-making as the core mechanisms through which individuals interpret suffering, reconstruct meaning, and sustain hope as they adjust to life on dialysis.

## Supplementary Information

Below is the link to the electronic supplementary material.

Supplementary file1 (DOCX 17 kb)

## Data Availability

The qualitative interview data supporting the findings of this study are **not publicly available** due to privacy and ethical restrictions. De-identified excerpts are available from the corresponding author upon reasonable request and with institutional ethics approval. Data are available, if necessary, with a formal request by contacting deniztasdemir@mehmetakif.edu.tr.
