# Supplementary material for: Faith, Meaning, and Life After Kidney Transplant Failure in Türkiye: A Qualitative Study of Spiritual Coping and Adjustment to Dialysis
Source: J Relig Health. 2026 Jun 24;65(5):5034–48. doi: 10.1007/s10943-026-02707-1 (PMC13649913; doi:10.1007/s10943-026-02707-1)
Supplement: Supplementary file 1 [file 10943_2026_2707_MOESM1_ESM.docx]

**Supplementary file: Participant Interview Guide**

1. Can you describe your experience when you learned that your kidney transplant had failed?

- How did you feel at that moment?
- What thoughts went through your mind?
- How did you make sense of what was happening?

2. What did losing the transplant mean to you personally?

- Did it affect how you see your health or your future?
- Did it influence your sense of independence?

3. How did you experience the transition back to dialysis?

- What was most challenging about returning to dialysis?
- If you had previous dialysis experience, how did this return compare?

4. In what ways has returning to dialysis affected your daily life?

- Physical well-being (e.g., energy, fatigue, discomfort)
- Daily routines
- Employment or financial situation

5. How has this experience affected you emotionally?

- Have your feelings changed over time?
- Have you experienced sadness, fear, anger, or hopelessness?

6. How has returning to dialysis affected your relationships?

- Family members
- Friends
- Social activities

7. Have you felt dependent on others during this period?

- How do you experience or interpret that dependency?

8. What has helped you cope with returning to dialysis?

- Personal strengths or beliefs
- Faith or spirituality
- Acceptance or adaptation strategies

9. What kinds of support have been most important to you?

- Family support
- Peer support (other patients)
- Healthcare professionals

10. Is there anything healthcare providers could do differently to better support patients in your situation?
11. How do you view your future now?

- Do you think about another transplant?
- Do you have concerns or uncertainties?

12. What gives you hope, if anything?
13. Has this experience changed how you define a “normal life”?

**Closing Question**

14. Is there anything else about your experience of returning to dialysis after transplant failure that you would like to share?
